# P-705. Non-Clinicians Effectively Perform Lung Ultrasound in Patients with Influenza-like Illness

**DOI:** 10.1093/ofid/ofae631.901

**Published:** 2025-01-29

**Authors:** Liam R Pauli, Claire S Wilson, Paul W Blair, Trishul Siddarthan, Phabiola Herrara, Shakir Hossen, Breana McBryde, Lauren Zimmerman, Gideon D Avornu, Richard E Rothman, Bhakti Hansoti, Tiffany Fong

**Affiliations:** Johns Hopkins University School of Medicine, Baltimore, Maryland; Johns Hopkins University School of Medicine, Baltimore, Maryland; Division of Infectious Diseases, Vanderbilt University Medical Center, Nashville, Tennessee; Division of Pulmonary and Critical Care Medicine, University of Miami, Miami, FL; Division of Pulmonary and Critical Care Medicine, Johns Hopkins University School of Medicine, Baltimore, MD, Miami, Florida; Division of Pulmonary and Critical Care Medicine, University of Miami, Miami, FL, Miami, Florida; University of Miami, Palmetto Bay, Florida; Johns Hopkins University School of Medicine, Baltimore, Maryland; Johns Hopkins University School of Medicine, Baltimore, Maryland; Johns Hopkins University School of Medicine, Baltimore, Maryland; John Hopkins University, Baltimore, Maryland; Department of Emergency Medicine, Johns Hopkins University, Baltimore MD, Baltimore, Maryland; Division of Pulmonary and Critical Care Medicine, University of Miami, Miami, FL, Miami, Florida

## Abstract

**Background:**

Point-of-care ultrasound (POCUS) is a low-cost, portable imaging modality with greater sensitivity than chest x-ray to evaluate acute respiratory infections including influenza-like illness (ILI). However, clinical scale has been limited by the availability of proficient operators and unclear training requirements. We aim to assess the feasibility of implementing study coordinator-performed lung POCUS into a longitudinal study of patients with ILI.

**Methods:**

From November 2021 to December 2023, we conducted an observational study to characterize the pathophysiology of ILI among outpatients. Patients were recruited from two urban academic hospital emergency departments and affiliated clinics. Study coordinators were trained through a 4-hour didactic session reviewing ultrasound physics and lung anatomy and pathology, together with a hands-on training session. Peer proctoring of two POCUS enrollment encounters followed. POCUS lung scans were obtained from patients by study coordinators at their enrollment visit (Day 0) and follow-up visits (Day 7, 14, 28). Scans were evaluated by blinded expert image reviewers. Data was analyzed using descriptive statistics including the proportion of images deemed technically adequate.

**Results:**

Fifteen study coordinators without previous ultrasound experience completed POCUS training over the course of the study. Using a standardized 12-zone imaging protocol, a total of 5311 POCUS lung scans were collected from 340 encounters. POCUS images were reviewed by study physicians and 100% of studies were considered adequate for diagnosis, with 96.0% of individual lung ultrasound clips judged to be technically adequate.

**Conclusion:**

Our study demonstrated that after a 4-hour training session, non-clinical research staff could successfully acquire protocolized lung POCUS images during a 2-year study to longitudinally monitor patients with ILI. This supports the feasibility of implementing POCUS into infectious diseases research and demonstrates the potential effectiveness for training new POCUS users to assess ILI.

**Disclosures:**

**Richard E. Rothman, MD, PhD**, abbvie: Grant/Research Support|CEPHEID: Advisor/Consultant|CEPHEID: Grant/Research Support|CEPHEID: Honoraria

